# Descriptive epidemiology of the injuries caused by the Chaharshanbeh Soori ceremony in Fars province in 2022

**DOI:** 10.5249/jivr.v17i1.1848

**Published:** 2025-01

**Authors:** Fatemeh Jafari, Mehrab Sayadi, Aboubakr Jafarnezhad, Hamed Karami, Fariba Moradi Ardekani, Pouria Azami

**Affiliations:** ^*a*^ Student Research Committee, Shiraz University of Medical Sciences, Shiraz, Iran.; ^*b*^ Non-Communicable Diseases Research Center, Shiraz University of Medical Sciences, Shiraz, Iran.; ^*c*^ School of Medicine, Shiraz University of Medical Sciences, Shiraz, Iran.

**Keywords:** Events, Injury, Chaharshanbeh Soori, Fireworks

## Abstract

**Background:**

Fireworks are widely used worldwide for their visual and auditory appeal. However, they pose significant safety risks, particularly to children and young adults, many of whom sustain serious injuries from exposure to these pyrotechnic devices. Given the preventable nature of such incidents and their associated challenges, this study aims to examine the epidemiological impact of injuries caused by fireworks during the Chaharshanbeh Soori ceremo-ny in Fars Province.

**Methods:**

This cross-sectional study included all individuals who experienced injuries between March 6, 2022, and April 4, 2022. Data were collected by emergency technicians affiliated with Shiraz University of Medical Sciences. Statistical analyses included descriptive statistics (mean ± standard deviation [SD] and frequency [%]) and analytical methods, specifically the chi-square test.

**Results:**

A total of 79 individuals were injured, with a mean age of 19.4 ± 11.5 years. The majority of the injured were male (73, 92.4%). Most injuries (66, 83.5%) occurred unintentionally, and 9 patients (11.4%) reported headache as a symptom, while 7 patients (8.9%) experienced visual disorders. Among those who received treatment, eye injuries were the most common (17, 26.2%). A significant difference was observed in recovery based on the injured body part; hand injuries had the highest recovery rate (6, 42.9%), compared to other injuries (p less than 0.029).

**Conclusions:**

The findings indicate that fireworks-related accidents during the Chaharshanbeh Soori ceremony predominantly affect teenagers and young adults, often occurring unintentionally. Given the significant physical, financial, and long-term consequences of these injuries, this study's results can inform policymakers in implementing preventive measures. Additionally, it highlights the need to raise awareness among the public and relevant authorities to ensure safer conduct of such ceremonies in the future.

## Introduction

Fireworks are widely used worldwide for their visual and auditory effects, playing an integral role in national and cultural celebrations.^1^ These include events such as the Fourth of July and New Year's Eve in the United States,^2^ Halloween and Guy Fawkes Day in the United Kingdom,^1^ the Chinese New Year, Diwali in India,^3^ and Milad Hazrat Muhammad in Libya.^4^ Despite the educational efforts through various media channels—such as newspapers, magazines, television, posters, pamphlets, and booklets-many individuals continue to face significant risks due to inadequate safety measures during the production and use of fireworks.^3^ Reports from numerous countries, including Australia, China, Hong Kong, Denmark, Finland, France, India, Israel, Italy, Japan, Lesotho, Libya, Malaysia, the Netherlands, New Zealand, the Philippines, Russia, Spain, Sweden, and the United Kingdom, underscore the global nature of this issue.^1,5,6^ The use of homemade tools or explosives often results in burns and other injuries. The hands are the most commonly injured body part, accounting for up to 53% of cases, followed by the eyes, which suffer the most severe injuries, representing up to 27%. In the United States, it is estimated that more than 12,000 individuals seek treatment in emergency departments annually due to firework-related injuries.^1^ Additionally, in 2005, fireworks were responsible for approximately $39 million in direct property damage in the United States^7^

The history of the Syrian Wednesday, or Persian Wednesday Night celebration, dates back to 1725 BC. This ancient Iranian festival is held at the end of the solar year, specifically on the last Tuesday night. In recent times, the festivities have increasingly involved the use of illegal or homemade explosives by young people.^8^ Each year, numerous individuals, particularly children and adolescents, sustain severe injuries from fireworks and related devices, often resulting in permanent disabilities such as amputations or blindness, which impose a significant burden on both families and society.^9^ A 2017 study reported 1,817 injuries, the majority of which occurred in Tehran and the northwest region.^10^ Another study in Iran indicated that 77% of injuries were caused by illegal homemade grenades.^11^ Furthermore, a study examining 11 years of data found that the average age of those injured was 18 years; of the 164 individuals, 145 were men, with 7 cases of amputation and 6 fatalities.^12^

The first step in effective policymaking to prevent and reduce the injuries and societal burden caused by fireworks during the Chaharshanbeh Soori celebration is to identify the specific areas, causes, and atrisk groups associated with such events. Although numerous injuries occur during the Chaharshanbeh Soori ceremony, existing studies have primarily focused on regions where the tradition is most prevalent, with limited up-to-date data available. Therefore, this study aims to investigate the epidemiological patterns of injuries associated with the Chaharshanbeh Soori celebration in Fars Province, to inform the development of targeted strategies for injury reduction and improved safety during this cultural event.

## Methods 

The present study was a cross-sectional investigation that included all individuals injured during the Chaharshanbeh Soori event between March 6, 2022, and April 4, 2022. Data were collected using a census approach. A structured checklist was used as the data collection instrument, which included demographic information, identification of at-risk populations, types of injurious agents, affected body parts, injury severity, symptoms observed during the initial assessment, primary causes of injuries, and injury outcomes. The data were gathered through face-to-face interviews conducted by emergency technicians affiliated with Shiraz University of Medical Sciences.

In this study, descriptive statistical methods were employed, including the calculation of means and standard deviations for quantitative data, and frequencies (n, %) for qualitative variables. The chi-square test was used for analytical purposes. A significance level of 0.05 was set for all statistical tests. Data analysis was conducted using SPSS software, version 26.

## Results

Among the 79 individuals who sustained injuries during the Chaharshanbeh Soori ceremony, 73 (92.4%) were male, and 67 (84.8%) resided in urban areas. The average age of the participants was 19.4 ± 11.5 years, and they had completed an average of 8 years of education (Table 1).

**Table 1:** Demographic Characteristics of Injured Individuals During the Chaharshanbeh Soori Ceremony in 2022

Variables	Number (%)
**Age (mean± sd) (year)**	19.4 ± 11.5
**Number of years of education (mean± sd)**	8.4 ± 4.6
**Sex**	
Male	73 (92.4)
Female	6 (7.6)
**Job**	
Child	4 (5.1)
Student	47 (59.5)
Student	9 (11.4)
other	19 (24.1)
**Living place**	
Urban	67 (84.8)
Rural	12 (15.2)

The results revealed that 53 (67.1%) of the incidents occurred on the street, with an average time of 18:00 ± 4.2 hours. Of these, 66 (83.5%) were unintentional. Regarding the type of explosive material, non-manmade explosives were the leading cause, accounting for 39.2% of the events (Table 2).

**Table 2:** Characteristics of Events During the Chaharshanbeh Soori Ceremony in 2022

Variables	Number (%)
**Time of the event (mean± sd) (hour)**	18± 4.2
**The place of the event**	
Home	13 (16.5)
Street	53 (67.1)
Other	13 (16.5)
**The situation of the event**	
During construction	14 (17.7)
During the game	8 (10.1)
During throw	32 (40.5)
During transportation	7 (8.9)
Other	18 (22.8)
**The person who caused the event**	
Self	50 (63.3)
Friends	9 (11.4)
other	20 (25.4)
**Substances causing the accident**	
Oil and gasoline	7 (8.9)
Gunpowder	5 (6.3)
Handmade chemicals	22 (27.8)
Non-manmade explosives	31 (39.2)
Other	14 (17.7)
**Type of fuel**	
Oil	6 (7.6)
Other	73 (92.4)
**The main cause of the event**	
Intentional	3 (3.8)
Unintentional	66 (83.5)
As a joke	10 (12.7)

**Figure 1:**
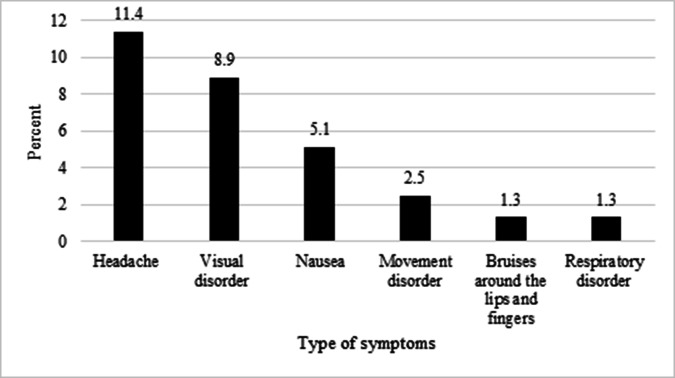
Percentage of Symptoms Noted During the First Visit by an Emergency Expert in Chaharshanbeh Soori Ceremony Events in 2022.

According to Figure 1, during the initial assessment by the emergency expert, 9 (11.4%) individuals reported headaches, 7 (8.9%) had visual disturbances, and 4 (5.1%) experienced nausea. Regarding treatment, 72 (89.9%) individuals received care either on an outpatient basis, at the scene of the accident, or in the emergency room, while 7 (8.9%) required hospitalization.

**Figure 2:**
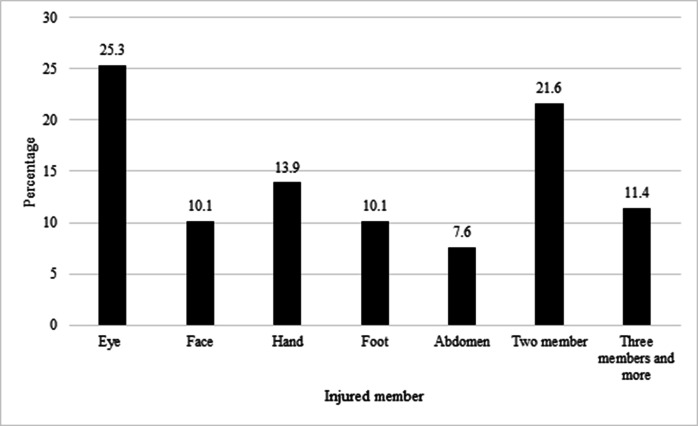
Percentage of Injuries by Body Part During the Chaharshanbeh Soori Ceremony in 2022.

In terms of the affected body parts, the eye sustained the highest frequency of injuries, with 20 cases (25.3%). The second and third most frequently injured body parts were the face, with 19 cases (24.1%), and the hand, with 11 cases (13.9%) (Figure 2).

Based on the findings presented in Table 3, 55 individuals (84.6%) who experienced unintentional injuries sought treatment and successfully recovered, compared to 11 individuals (78.6%) with intentional injuries (p<0.0001). Additionally, eye injuries represented the largest percentage of cases among those who received treatment (17 individuals, 26.2%), while hand injuries had the highest recovery rate in the recovery group (6 individuals, 42.9%) (p<0.029).

**Table 3:** Relationship Between Injury Status, Event Causes, and Affected Body Parts During the Chaharshanbeh Soori Ceremony in 2022.

Variable	The status of the injured person		p-value
	Getting treatment	Full recovery	
**The main cause of the event**			<0.0001
Intentional	0 (0%)	3 (21.4%)	
Unintentional	55 (84.6%)	11 (78.6%)	
As a joke	10 (15.4%)	0 (0%)	
Injured member			0.029
Eye	17 (26.2%)	3 (21.4%)	
Face	8 (12.3%)	0 (0%)	
Hand	5 (7.7%)	6 (42.9%)	
Foot	8 (12.3%)	0 (0%)	
Abdomen	5 (7.7%)	1 (7.1%)	
Two member	14 (21.5%)	3 (21.4%)	
Three members and more	8 (12.3%)	1 (7.1%)	

The latest analytical results revealed that 59 individuals (90.8%) who received treatment resided in urban areas, compared to 6 individuals (9.2%) from rural are-as (p = 0.001). Additionally, 60 individuals (90.9%) who experienced unintentional injuries lived in the city, while among those who sustained injuries intentionally or as part of a joke, 3 individuals (100%) and 3 individuals (30%) were from rural areas, respectively (p<0.001) (Table 4).

**Table 4:** Relationship Between Residence Location, Injury Status, and Event Causes During the Chaharshanbeh Soori Ceremony in 2022.

Variable	Living place		p-value
	Urban	Rural	
**The status of the injured person**			0.001
Getting treatment	59 (90.8%)	6 (9.2%)	
Full recovery	8 (57.1%)	6 (42.9%)	
**The main cause of the event**			<0.001
Intentional	0 (0%)	3 (100%)	
Unintentional	60 (90.9%)	6 (9.1%)	
As a joke	7 (70%)	3 (30%)	

## Discussion

The Chaharshanbeh Soori ceremony, celebrated on the last Wednesday before Nowruz, has increasingly been associated with significant injuries and fatalities due to the use of fireworks and other incendiary devices. A report from 2023 indicated that at least 26 individuals died and 4,000 were injured during the celebrations, with 880 suffering eye injuries and 850 experiencing varying degrees of burns.^13^ Another study found that approximately 83% of firecracker-related injuries involved men under the age of 30.^14^ Additionally, a study conducted in Tehran documented that nearly half of the 35 patients treated for firework-related injuries had lacerations or cuts, with hands and faces being the most common injury sites.^9^ Although the ceremony, rooted in Zoroastrian traditions, symbolizes purification and the welcoming of spring, research suggests that the evolution of these customs has contributed to increasingly dangerous practices involving fireworks and explosives.^15,16^ From a psychological perspective, studies indicate that young individuals may perceive themselves as invulnerable during such celebrations, leading to a disregard for safety warnings issued by authorities.^17^

The findings from the current study indicate that the majority of injuries associated with the Chaharshanbeh Soori ceremony were sustained by men. This is likely due to the greater involvement of males in the fire-works activities, which are deeply rooted in cultural and societal customs that may be viewed as a favored and traditional practice for men. The socio-cultural context of Iranian society has contributed to higher male participation in such events.^18,19^ Furthermore, the types of activities associated with Chaharshanbeh Soori may inherently appeal to men, as curiosity regarding risks and dangerous activities is often more pronounced in boys than in girls. Physiological and personality factors could also play a significant role, underscoring the need for further comprehensive research.^20^

The findings of this study revealed that the highest number of injuries related to the Chaharshanbeh Soori festivities occurred among residents of urban areas. Characteristics of urban environments, such as expansive public spaces designed for recreation and leisure—like parkstend to attract larger numbers of people, facilitating widespread participation in various events, including fireworks displays. One study indicated that urban areas, with their greater cultural diversity, are more conducive to hosting cultural and religious celebrations than rural areas.^21^

The findings of this study indicated that the mean education level among participants in the Chaharshanbeh Soori event was eight grades, which aligns with the findings of Vaghardoost et al.^12^ A possible explanation for this trend is that individuals with lower education levels often seek to enhance their social standing by engaging in such festivities. For many from this demographic, enjoyment and leisure are significant factors that drive their participation.

The results indicated that individuals who were accidentally injured experienced the most recovery and treatment. While it cannot be definitively stated that there is a significant correlation between the intentional and unintentional causes of fire-related injuries and recovery, several potential explanations can be considered. One possible explanation is the apprehension regarding the legal repercussions of using fireworks. Specifically, deliberately starting a fire can lead to legal consequences, and if discovered, it is classified as a crime against public safety. As a result, individuals who intentionally set fires may attempt to conceal the cause of their injury due to fear of legal consequences. Even when facing burns or severe harm, they may neglect the treatment process, which can disrupt their recovery. This issue was also highlighted in a study by Green et al., which explored the patterns and motivations behind arson^22^ and is supported by other studies.^23,24^ Mental health issues, such as personality disorders, depression, and anxiety, are among the factors identified by researchers that may contribute to harmful behaviors and hinder recovery.^24-26^ However, further research is needed to establish a meaningful relationship between intentional and accidental fire injuries and treatment outcomes.

The findings of this research indicated that the most affected body parts were the upper limbs, including the eyes, hands, and face, with occasional injuries involving multiple areas. Other studies have also reported the hands as the most commonly injured body part.^12,27^ Additionally, we found that eye injuries accounted for the highest percentage among those who received treatment, while hand injuries had the highest frequency in the recovery group. Given that the eye is a more sensitive and vulnerable organ, it is crucial to place greater emphasis on preventing eye injuries and promoting early treatment. Overall, effective treatment and recovery from injuries caused by fireworks and fires require careful attention to the type and severity of the injury, as well as the application of appropriate treatment methods.^28-30^ Furthermore, a lack of understanding regarding the severity of injuries, particularly burns, can hinder recovery and treatment, potentially leading to more serious complications.

The results showed that the majority of individuals seeking medical attention were urban residents, indicating that those receiving treatment predominantly came from city areas. Distinct differences were observed between urban and rural incidents related to Chaharshanbeh Soori, as well as in subsequent recovery and treatment. It can be inferred that in large cities, the higher population density and closer proximity of buildings increase the risk of fire spreading, leading to greater harm and more extensive damage. Additionally, the presence of smoke, gases, and combustible particles in the urban air—resulting from industrial activity, vehicular traffic, and construction materials—can exacerbate fire hazards. The prevalence of tall buildings and skyscrapers also limits air circulation, which may negatively impact recovery and treatment for urban residents. Traffic congestion and the time it takes for injured individuals to access medical care centers are additional contributing factors. In contrast, rural areas may experience lower risks due to the greater distances between structures and the availability of open spaces. The lower population density in rural areas, along with fewer factories and skyscrapers using flammable materials, further supports this observation. Our findings align with previous research, which highlights these trends.^31-33^

A key strength of this research is its provision of up-to-date information on Chaharshanbeh Soori events, an area that has received limited attention from researchers. However, a limitation of this study is the scarcity of existing research on Chaharshanbeh Soori, which restricts the ability to compare findings with other studies. Additionally, the data collected may be incomplete or limited, as many individuals opt for home remedies to treat their injuries. Furthermore, the results may only apply to a specific region or age group, and more comprehensive studies are needed to enhance the generalizability of the findings.

## Conclusion

The importance of the Chaharshanbeh Soori ceremony in Iran, with its deep cultural, historical, and social significance, is clear. Its national and ancient roots, especially among teenagers and young people, make this celebration an important part of Iranian society. This research helps to better understand the culture and history of the celebration, as well as its impact on society. Since Chaharshanbeh Soori is an annual event, future research should focus on longitudinal studies to track injury trends and explore the social and psychological factors that contribute to risky behaviors among participants. Comparative studies could also examine the impact of different cultural practices on injury rates. Public health interventions should aim to increase awareness, promote safer celebration alternatives, and collaborate with local authorities to enforce stricter regulations on fireworks. Additionally, providing adequate medical care and preparing individuals for potential injuries will further enhance safety during the celebration.

**Acknowledgement**

We are very grateful to the professors, honorable advisers and everyone who helped us in this research.

**Authors’ Contribution:** FJ: Formal analysis, Writing - Original Draft, Visualization; MS: Conceptualization, Methodology; AJ: Writing – Review & Editing, Software; HK: Investigation, Resources, Data Curation; FM: Project administration, Funding acquisition; PA: Resources, Writing - Review & Editing
